# TRPC1 channels underlie stretch-modulated sarcoplasmic reticulum calcium leak in cardiomyocytes

**DOI:** 10.3389/fphys.2022.1056657

**Published:** 2022-12-23

**Authors:** Molly E. Streiff, Andrea C. Corbin, Azmi A. Ahmad, Chris Hunter, Frank B. Sachse

**Affiliations:** ^1^ Nora Eccles Harrison Cardiovascular Research and Training Institute, University of Utah, Salt Lake City, UT, United States; ^2^ Department of Biomedical Engineering, University of Utah, Salt Lake City, UT, United States

**Keywords:** TRPC1, cardiomyocyte, calcium, sarcoplasmic reticulum, mechanosensitivity

## Abstract

Transient receptor potential canonical 1 (TRPC1) channels are Ca^2+^-permeable ion channels expressed in cardiomyocytes. An involvement of TRPC1 channels in cardiac diseases is widely established. However, the physiological role of TRPC1 channels and the mechanisms through which they contribute to disease development are still under investigation. Our prior work suggested that TRPC1 forms Ca^2+^ leak channels located in the sarcoplasmic reticulum (SR) membrane. Prior studies suggested that TRPC1 channels in the cell membrane are mechanosensitive, but this was not yet investigated in cardiomyocytes or for SR localized TRPC1 channels. We applied adenoviral transfection to overexpress or suppress TRPC1 expression in neonatal rat ventricular myocytes (NRVMs). Transfections were evaluated with RT-qPCR, western blot, and fluorescent imaging. Single-molecule localization microscopy revealed high colocalization of exogenously expressed TRPC1 and the sarco/endoplasmic reticulum Ca^2+^ ATPase (SERCA2). To test our hypothesis that TRPC1 channels contribute to mechanosensitive Ca^2+^ SR leak, we directly measured SR Ca^2+^ concentration ([Ca^2+^]_SR_) using adenoviral transfection with a novel ratiometric genetically encoded SR-targeting Ca^2+^ sensor. We performed fluorescence imaging to quantitatively assess [Ca^2+^]_SR_ and leak through TRPC1 channels of NRVMs cultured on stretchable silicone membranes. [Ca^2+^]_SR_ was increased in cells with suppressed TRPC1 expression vs. control and Transient receptor potential canonical 1-overexpressing cells. We also detected a significant reduction in [Ca^2+^]_SR_ in cells with Transient receptor potential canonical 1 overexpression when 10% uniaxial stretch was applied. These findings indicate that TRPC1 channels underlie the mechanosensitive modulation of [Ca^2+^]_SR_. Our findings are critical for understanding the physiological role of TRPC1 channels and support the development of pharmacological therapies for cardiac diseases.

## 1 Introduction

Transient receptor potential canonical 1 (TRPC1) forms non-selective cation channels expressed in cardiomyocytes. TRPC1 channels are highly permeable to Ca^2+^, an important second messenger vital to a diverse range of cellular functions including signaling pathways such as excitation-contraction (EC) coupling. Ca^2+^ is also a critical player in adverse cardiac remodeling and heart failure. The involvement of TRPC channels in cardiac diseases has been reviewed extensively (Guinamard and Bois, 2007; Nishida and Kurose, 2008; Watanabe et al., 2008; Rowell et al., 2010; Eder and Molkentin, 2011; Smani et al., 2015; Yue et al., 2015; Ahmad et al., 2017; Eder, 2017; Xiao et al., 2017; Vennekens, 2018; Falcón et al., 2019; Falcón et al., 2020). TRPC1 is well-established to play a critical role in the development of cardiac diseases including heart failure and hypertrophy. Maladaptive cardiac hypertrophy has been linked to elevated expression of TRPC1 following endothelin-1 treatment in neonatal rat ventricular myocytes (NRVMs) (Ohba et al., 2007; Kiso et al., 2013) and following isoproterenol injections in adult rat hearts (Chen et al., 2013). In addition, upregulation of TRPC1 in hypertrophy was demonstrated in pressure overload models including abdominal aortic-banded rats (Ohba et al., 2007), murine models of isolated left and right ventricular pressure overload *via* thoracic aortic and pulmonary arterial constriction, respectively (Morine et al., 2016), and mice subjected to pressure overload induced by transverse aortic constriction (Seth et al., 2009). In rats with hyperthyroidism-induced cardiac hypertrophy, increased TRPC1 activation was linked to the deterioration of Ca^2+^ balance contributing to the hypertrophic remodeling (Bektur Aykanat et al., 2021). Importantly, the upregulation of TRPC1 in cardiac disease was also found in human heart samples. Increased mRNA levels of TRPC1 were measured in end-stage heart failure in both left and right ventricles compared to non-failing left and right ventricles, respectively (Morine et al., 2016). Expression of TRPC1 was elevated in patients with hypertrophic cardiomyopathy and heart failure compared to healthy donor hearts (Tang et al., 2019). Embryonic stem cell-derived cardiomyocytes treated with phorbol 12-myristate 13-acetate also exhibited hypertrophy and a higher level of TRPC1 expression compared to control (Tang et al., 2019). Induced pluripotent stem cell-derived cardiomyocytes from hypertrophic cardiomyopathy patients exhibited diastolic dysfunction at the cellular level and increased expression of TRPC1 (Wu et al., 2019). Conversely, overexpression of TRPC1 induced cardiomyocyte hypertrophy, showing that TRPC1 is sufficient to induce cardiac hypertrophy (Tang et al., 2019).

Blocking or suppressing the expression of TRPC1 was sufficient to attenuate cardiac hypertrophy. Silencing of TRPC1 in NRVMs with siRNA has been shown to prevent cardiac hypertrophy induced by endothelin-1, angiotensin-II, and phenylephrine (Ohba et al., 2007). Mice with TRPC1 gene deletion maintained preserved cardiac function and failed to manifest evidence of maladaptive cardiac hypertrophy following pressure overload-induced hypertrophy *via* transverse aortic constriction (Seth et al., 2009). In human pluripotent stem cell lines with TRPC1 knockout by CRISPR/Cas9, cardiomyocyte hypertrophy phenotype induced by phorbol 12-myristate 13-acetate was significantly attenuated (Tang et al., 2019). These studies highlight why TRPC1 channels are regarded as promising targets for therapeutic interventions for maladaptive cardiac remodeling.

TRPC1 channels are mediators of pathological cardiac remodeling. However, the mechanisms by which TRPC1 contributes to maladaptive cardiac remodeling in disease are yet to be elucidated. TRPC1 channels are commonly described as store-operated channels (Brazer et al., 2003; Parekh and Putney, 2005; Ju and Allen, 2007; Ong et al., 2007; Liao et al., 2008; Saleh et al., 2008; Cheng et al., 2011; Cheng et al., 2013; Sabourin et al., 2015; Ong et al., 2016; Sabourin et al., 2016; Eder, 2017; Wen et al., 2018). TRPC1 channels were also first suggested as candidates for stretch-activated channels in frog oocytes (Maroto et al., 2005). Evidence for a role of TRPC1 channels in mechanotransduction was provided in sensory neurons (Alessandri-Haber et al., 2009; Staaf et al., 2009; Garrison et al., 2012) as well as endothelial cells (Berrout et al., 2012) and epithelioid cells (Yu and Li, 2017). However, mechanosensitivity of TRPC1 was also disputed in smooth muscle cells (Dietrich et al., 2007), African green monkey kidney cells, and Chinese hamster ovary cells (Gottlieb et al., 2008). Mechanosensitivity of TRPC1 is still ill-defined in cardiomyocytes. Most studies assumed TRPC1 channels are located on the sarcolemma, contributing to Ca^2+^ influx from the extracellular fluid. However, we recently provided evidence that the channels are located in the membrane of the sarcoplasmic reticulum (SR), a crucial Ca^2+^ store for EC coupling in cardiac myocytes, and function as Ca^2+^ leak channels (Hu et al., 2020). A similar location and role of TRPC1 channels have been suggested for skeletal muscle cells (Berbey et al., 2009).

Here, we provide additional evidence that TRPC1 channels form SR Ca^2+^ leak channels. We tested the hypothesis that SR Ca^2+^ leak through TRPC1 channels is altered by mechanical stretch in cardiomyocytes. We used an SR-targeting Ca^2+^ sensor and implemented fluorescent imaging to directly evaluate SR Ca^2+^ concentrations in NRVMs with overexpressed or suppressed TRPC1. Adenoviral transfection to alter TRPC1 expression in NRVMs was extensively assessed using qPCR, western blot, and imaging. Our studies provide the first evidence for a role of TRPC1 in mechanosensitivity in cardiomyocytes. We suggest TRPC1 channels are involved in stretch-modulated SR Ca^2+^ leak in cardiomyocytes. This function points at a mechanism in maladaptive cardiac remodeling by modulating SR Ca^2+^ handling under pathological strain conditions. Further explorations on the mechanics of TRPC1 channel function will be critical for the development of drug modulations for maladaptive cardiac remodeling targeting TRPC channels.

## 2 Materials and methods

All studies were reviewed by the Institutional Animal Care and Use Committee (#18-11006 approved on 28 November 2018 and #19-02012 approved on 05 June 2019) and performed at the University of Utah in accordance with National Institute of Health Guidelines for the Care and Use of Animals.

### 2.1 NRVM culture and infection

Ventricular myocytes were isolated from 1-day old rats (NCIS, Worthington Biochemical Corporation, Lakewood, NJ, United States). NRVMs were cultured in Nunc™ Lab-Tek™ II chambered coverglass (155409, Thermo Fisher Scientific) treated with fibronectin for structural imaging, stretchable silicon chambers (STB-CH-0.02, STREX Inc., Kita-Ku, Osaka, Japan) treated with fibronectin for fluorescent Ca^2+^ imaging, or tissue culture plates. At 24 h post-plating, custom adenoviruses were applied to transfect NRVMs with TRPC1-TagBFP2 (VB191113-1698mzn, Vector Builder, Chicago, IL, United States), TagBFP2 (VB191113-1704zna), shRNA-TRPC1-TagBFP2 (VB191230-1471zkf), or scrambled shRNA-TagBFP2 (VB191230-1721wmw). Additionally, a custom adenovirus was applied to transfect the SR-targeting genetically encoded Ca^2+^ sensor GCEPIAer tagged with SNAP (Vector Biolabs, Malvern, PA, United States). The GCEPIAer-SNAP plasmid was developed and provided to us by (Luo et al., 2019). At the time of infection and for all subsequent steps, 10 μg/ml cytosine-β-D-arabinofuranose hydrochloride (J65671, Thermo Fisher Scientific, Waltham, MA, United States) was added to the culture media to inhibit fibroblast proliferation. Adenoviruses were washed out 24 h post-infection and NRVMs were studied 24 h post-washout. For RT-qPCR, neonatal mouse ventricular myocytes (NMVMs) were isolated from 1-day old mice (Pierce™ Primary Isolation Kit, 88281, Thermo Fisher Scientific).

### 2.2 Western blot

Wild type (WT) and NRVMs infected with TagBFP2, TRPC1-TagBFP2 or shRNA-TRPC1-TagBFP2 were lysed in RIPA buffer. Protein was collected and protein concentration was quantified with the Pierce BCA protein assay kit (23227, Thermo Fisher Scientific). Samples containing 2 μg/μl protein lysate with radio immunoprecipitation assay (RIPA) buffer, 2-Mercaptoethanol, and 6× loading dye were heated to 70°C for 10 min for protein reduction. 35 µg protein was loaded on a 4%–12% Bis-Tris Plus Gel (NW04120BOX, Thermo Fisher Scientific) and electrophoresed in MOPS running buffer (B001, Invitrogen) at 200 V for 35 min. Protein was transferred onto a 0.45 μm nitrocellulose membrane in Tris-Glycine-Methanol buffer for 1 h at 250 mA. The transferred blot was blocked for 1 h at room temperature in a solution of 5% BSA in Tris-Buffered Saline with Tween 20 (TBS-T). Primary antibodies for tRFP (AB233, Evrogen) and GAPDH (AB8245, Abcam) were applied at a dilution of 1:2,500 in blocking solution for 1.5 h. Secondary antibodies (AB97069 and AB97040, Abcam) and Precision Protein StrepTactin-HRP (1610380, Bio-Rad) were applied at 1:30,000 for 1 h. The blot was then incubated in Western Bright ECL HRP substrate kit (K012045, Advansta, San Jose, CA, United States) for 2 min and imaged on Bio-Rad Imager (Bio-Rad).

### 2.3 Reverse transcription quantitative real-time polymerase chain reaction (RT-qPCR)

RT-qPCR was performed on mRNA collected from NMVMs and NRVMs 48 h post-infection. Total RNA was extracted with either RNeasy^®^ Plus Mini Kit (74134, Qiagen, Hilden, Germany) or Direct-zol™ RNA MiniPrep Plus (R2071, Zymo Research, Irvine, CA, United States) with TRI-Reagent^®^ (R2050-1-50, Zymo Research) following the instructions of the manufacturers. The quantity, purity, and integrity of RNA stocks were determined by spectrophotometry (NanoDrop Lite, Thermo Fisher Scientific) (Supplementary Table S1). cDNA synthesis was carried out using the SuperScriptTM VILO^TM^ cDNA Synthesis Kit (11754-050 and 11754-250, Thermo Fisher Scientific) according to the manufacturer supplied protocol. The quantity, purity, and integrity of cDNA were also determined by spectrophotometry (Supplementary Table S1). RT-qPCR of cDNA samples was performed in triplicate with QuantStudio™12 K Flex Real-Time PCR System from Applied Biosystems (Thermo Fisher Scientific). TaqMan™ primer for 18S (Rn03928990_g1), Thermo Fisher Scientific) was used as endogenous internal control. We applied five TaqMan™ primers, with FAM-MGB dye, for TRPC1 (Rn00677552_m1, Rn00677554_m1, Rn00585625_m1, Rn00677549_g1, Rn01447000_m1, Thermo Fisher Scientific) and used the TaqMan^®^ Fast Advanced Master Mix (Thermo Fisher Scientific) with a total volume of 10 µl. The following cycling profile was applied: 2 min at 50°C, followed by incubation for 2 min at 95°C. For amplification, 40 cycles were performed with incubation at 95°C for 1 s, followed by 60°C for 20 s for annealing. Relative expression values and fold changes were calculated using the ∆∆Ct analysis method with 18 s as the reference gene and WT rat as the control sample. Fold change was measured as 2^−ΔΔCT^.

### 2.4 Single-molecule localization microscopy

NRVMs infected with TRPC1-TagBFP2 or TagBFP2 were fixed for 15 min at room temperature in 1% paraformaldehyde (PFA) 48 h post-infection. Cells were then permeabilized for 18 min in 0.25% Triton X-100 (VWR International, Radnor, PA, United States). Image-iT Fx Signal Enhancer (I36933, Thermo Fisher Scientific) was applied for 30 min, followed by blocking in 10% normal donkey serum (D9663, Millipore, Billerica, MA, United States) for 60 min. NRVMs were then incubated in primary antibodies for tRFP (AB233, Evrogen) and SERCA2 (MA3-910, Thermo Fisher Scientific) overnight in 2% normal donkey serum at 4°C. Secondary antibodies Alexa Fluor 647 (A-31573, Thermo Fisher Scientific) and CF 568 (SAB4600075, Sigma-Aldrich) were applied at room temperature the following morning for 2 h.

During imaging, labeled NRVMs were immersed in a solution containing 20 mM MEA, 1% (*v*/*v*) 2-Mercaptoethanol, and an oxygen scavenging system (glucose oxidase and catalase) in a buffer of 50 mM Tris, 10 mM NaCl, and 10% (*w*/*v*) glucose. Three-dimensional single-molecule localization microscopy was performed using a Vutara 352 (Bruker Corporation, Middleton, WI, United States) equipped with a 60× silicone immersion objective (numerical aperture: 1.3). CF 568 was excited at 561 nm and collected at 600 ± 25 nm and Alexa Fluor 647 was excited at 640 nm, with an additional 405 nm activation laser, and collected at 690 ± 45 nm. We acquired image stacks of at least 5,000 frames with 20 ms exposure per signal. Reconstructed stacks were approximately 40 μm × 40 μm × 2 µm. Localizations greater than median radial precision and less than median photon count were filtered out for analysis. Co-localization analysis was performed using Vutara SRX software (version 6.05.35).

### 2.5 Fluorescent microscopy of un-stretched and stretched cells

Live NRVMs infected with TRPC1-TagBFP2, TagBFP2, shRNA-TRPC1-TagBFP2, or scrambled shRNA-TagBFP2 NRVMs were loaded with SNAP-Cell 647-SiR (S9102S, New England Biolabs) for 45 min at 37°C. Imaging was performed on a Zeiss microscope (Carl Zeiss, Jena, Germany) equipped with a 20× lens and a CMOS camera (Prime 95B, Teledyne Photometrics, Tucson, AZ, United States). TagBFP2 fluorescence was excited with 405 nm laser and collected with a 460 ± 25 nm filter. GCEPIAer fluorescence was excited with 470 nm laser and collected with 525 ± 25 nm filter. SNAP-Cell 647-SiR fluorescence was excited with 640 nm laser and collected with 700 ± 37.5 nm filter. NRVMs cultured on stretchable silicone membranes were either left at rest or statically stretched 10% using a programmable microscope-mountable uniaxial stretching system (STB-150W, STREX Inc.) shown in Supplementary Figure S1. The system applies stepper motors and stretching arms for stretching the silicone chambers. The chambers were attached to pins in the stretching arms. The system was programmed to stretch the silicone membranes 10% in 0.5 s and then hold the membrane in sustained stretch until the experiment was completed. Cells were electrically paced at 0.5 Hz. After 2–3 min of pacing, Ca^2+^ transients were collected for 30 s.

### 2.6 Calibration of Ca^2+^ concentrations from fluorescent signals

For calibration of the Ca^2+^ signals measured *via* fluorescent imaging, solutions were switched to a Ca^2+^ free solution (in mM: 126 NaCl, 4.4 KCl, 1 MgCl_2_, 24 HEPES, 11 D-Glucose, 12.5 NaOH, 10 EGTA, 40 2,3-Butanedione monoxime (BDM) and 0.5 probenecid) including 10 µm ionomycin (I0634, Sigma-Aldrich) to permeate cell membranes and then a solution containing 100 mM Ca^2+^ (in mM: 26 NaCl, 4.4 KCl, 1 MgCl_2_, 24 HEPES, 11 D-Glucose, 12.5 NaOH, 100 CaCl_2_, 40 BDM and 0.5 probenecid). SR Ca^2+^ concentration ([Ca^2+^]_SR_, µM) was calculated according to fitted Hill curves for Ca^2+^ titration from (Luo et al., 2019) using Eq. 1:

$$R=\frac{{{\left[{Ca}^{2+}\right]}_{SR}}^{1.26}}{{{\left[{Ca}^{2+}\right]}_{SR}}^{1.26}+514}\left(R_{\max}-R_{\min}\right)+R_{\min\;}$$ (1)

where ***R*** is the ratio of GCEPIAer/SNAP fluorescence in modified Tyrode solution, ***R*_*min*_** is the ratio in 0 **Ca^2+^** solution, and ***R*_*max*_** is the ratio in 100 **Ca^2+^** solution.

### 2.7 Image analysis and data extraction

For Ca^2+^ imaging experiments, image sequences were analyzed using programs written in MATLAB (R2020b). Briefly, background fluorescence was subtracted from the image stack from each signal using built-in MATLAB functions “multithresh” and “imquantize” to apply a threshold that separates background and cells. The median fluorescence value from all pixels in the background was subtracted from the entire image stack. Each cell was segmented roughly by manual circling and then thresholding to separate background and nuclei included in the manual roi. Data extraction was performed on traces from each threshold corrected cell region using built-in MATLAB function ‘find peaks’ to identify minimum and maximum points on the calcium transients corresponding to systolic and diastolic [Ca^2+^]_SR_. Slope of the transients’ downstroke was calculated by fitting a linear regression and the reuptake time constant was calculated by fitting an exponential curve.

### 2.8 Statistical analysis

Statistical analyses of qPCR results and super-resolution colocalization were performed in MATLAB (R2020b). Differences were evaluated using one-way analysis of variance (ANOVA) and Student’s unpaired *t*-test. Results are presented as mean ± SEM. Significance is indicated for *p* < 0.05.

For Ca^2+^ imaging experiments, statistical analyses were performed in Stata (BE 17.0). Regression diagnostics were performed and cooksd was used to identify and exclude influential observations. A multilevel mixed-effects linear regression was used to account for the true effect size based on cells measured on the same membrane; linear combinations were used to analyze statistical differences between groups. Supplementary Figure S2 presents the Stata code to provide details. Data are displayed in scatter plots showing one point per membrane representing the average of all cells measured on the membrane. Mean and SEM calculated by the multilevel mixed-effects model are represented by bars. All results are presented as mean ± SEM. Significance is indicated for *p* < 0.05.

## 3 Results

### 3.1 Confirmation of TagBFP2 adenoviral expression

**FIGURE 1:**
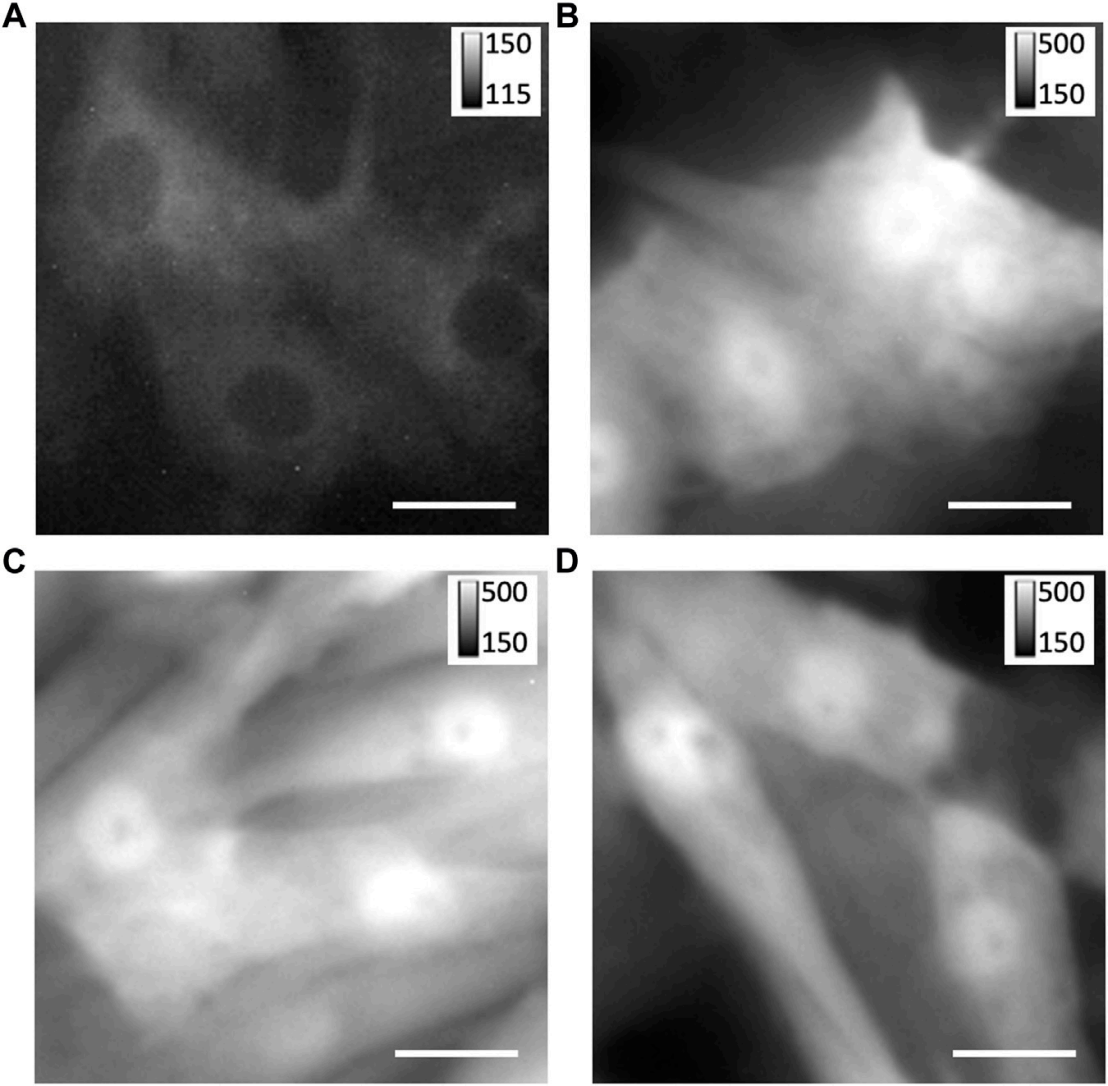
Expression of TagBFP2 adenoviral constructs in NRVMs. Fluorescent imaging of TagBFP2 in cells expressing **(A)** TRPC1-TagBFP2, **(B)** TagBFP2, **(C)** scrambled shRNA-TagBFP2, and **(D)** shRNA-TRPC1-TagBFP2. Scale bars = 20 µm.

We investigated custom adenoviral constructs for the transfection of TRPC1-TagBFP2, TagBFP2, shRNA-TRPC1-TagBFP2, and scrambled shRNA-TagBFP2. To confirm the successful transfection of our constructs, we applied fluorescent imaging, western blotting, and RT-qPCR. Fluorescent imaging of live cells expressing TagBFP2 revealed intracellular localization in TRPC1-TagBFP2 cells (Figure 1A). Nuclei lacked fluorescence as in other intracellular regions. Bright fluorescence filled the entirety of TagBFP2 control cells, scrambled shRNA-TagBFP2 cells, and shRNA-TRPC1-TagBFP2 cells (Figures 1B–D). To further confirm expression of the transfected proteins, we performed western blotting with the primary antibody anti-tRFP, which recognizes several fluorescent proteins including TagBFP2 (Supplementary Figure S3). Housekeeping gene GAPDH exhibits a clear band around 36 kDa in all samples (white arrow). In TagBFP2 control cells and shRNA-TRPC1-TagBFP2 cells, the TagBFP2 is expressed on its own and appears in our western blot around 20 and 27 kDa, matching expected bands for antibody specifications from the manufacturer. In TRPC1-TagBFP2 cells, a strong band appears at 96.2 kDa, corresponding to the sum of TRPC1 and TagBFP2.

Further, we measured mRNA expression of TRPC1 in our adenoviral constructs using RT-qPCR. The quantity, purity, and integrity of RNA stocks were determined by spectrophotometry (Supplementary Table S1). A profile of our endogenous control, 18 s, across samples and example amplification plots are provided in Supplementary Figure S4. Each sample had A260/A280 values between 1.99 and 2.07, showing high purity of RNA extraction. The quantity and purity of cDNA were also determined by spectrophotometry, and each sample had A260/A280 values between 1.86 and 1.92 (Supplementary Table S1). We evaluated 5 primers for rat TRPC1 for cross-reactivity with the mouse TRPC1 gene using WT NRVMs and WT NMVMs (Supplementary Table S2). Primers targeting rat TRPC1 exon 3-4 (Rn00677552_m1) and exon 10-11 (Rn00677549_g1) exhibited cross-reactivity with mouse, while the other three primers (Rn00677554_m1, Rn00585625_m1, Rn01447000_m1) appear to be specific to rat.

**FIGURE 2:**
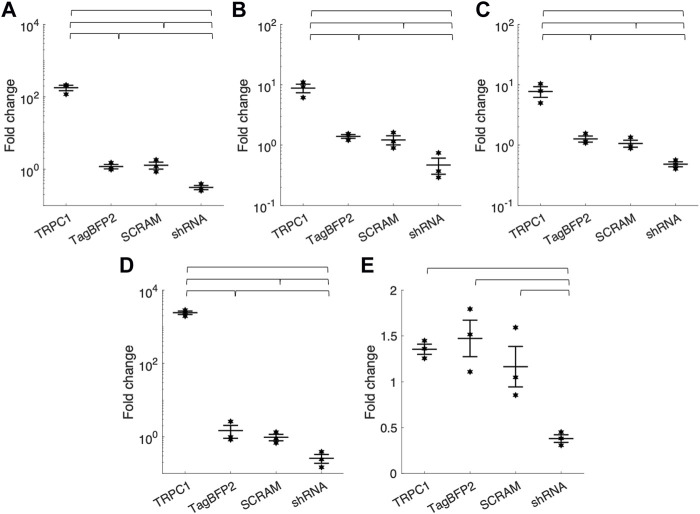
Mean fold change of TRPC1 mRNA expression of infected NRVMs from WT NRVMs quantified by RT-qPCR with primers targeting rat TRPC1 **(A)** exon 3-4 (Rn00677552_m1) **(B)** exon 6-7 (Rn00677554_m1) **(C)** exon 8-9 (Rn00585625_m1) **(D)** exon 10-11 (Rn00677549_g1) and **(E)** exon 11-12 (Rn01447000_m1) (*n* = 3 litters). Brackets mark significant differences (*p* < 0.05).

Table 1; Figure 2 show RT-qPCR results as the mean fold change of TRPC1 expression in each infected cell group compared to WT NRVMs for all five tested primers (*n* = 3 L). The two control groups, TagBFP2 and scrambled shRNA-TagBFP2, showed no significant difference in TRPC1 expression for any of the primers tested (*p* = 0.5–0.9). Mean fold change of TRPC1 expression compared to WT NRVMs is significantly greater in TRPC1-TagBFP2 cells than TagBFP2, scrambled shRNA-TagBFP2, and shRNA-TRPC1-TagBFP2 cells for the primer targeting exon 3-4 (Rn00677552_m1, all *p* < 0.001), the primer targeting exon 6-7 (Rn00677554_m1, all *p* < 0.001), the primer targeting exon 8-9 (Rn00585625_m1, all *p* ≤ 0.001) and the primer targeting exon 10-11 (Rn00677549_g1, all *p* < 0.001). Interestingly, the primer targeting rat TRPC1 exon 11-12 (Rn01447000_m1) was the only primer that didn’t show significantly increased expression in TRPC1-TagBFP2 cells compared to controls TagBFP2 and scrambled shRNA-TagBFP2 (*p* = 0.945 and *p* = 0.814, respectively). shRNA-TRPC1-TagBFP2 cells express significantly less TRPC1 than all other groups of NRVMs for each primer (all *p* < 0.05).

**TABLE 1:** Mean fold change of TRPC1 mRNA from WT NRVMs of all RT-qPCR primers.

Primer	Primer Region	TRPC1-TagBFP2	TagBFP2	Scrambled shRNA-TagBFP2	shRNA-TRPC1-TagBFP2
Rn00677552_m1^a^	Exon 3–4	178.49 ± 30.47	1.19 ± 0.17	1.29 ± 0.28	0.31 ± 0.04
Rn00677554_m1	Exon 6–7	8.80 ± 1.43	1.40 ± 0.10	1.22 ± 0.21	0.47 ± 0.14
Rn00585625_m1	Exon 8–9	7.74 ± 1.55	1.27 ± 0.15	1.07 ± 0.14	0.48 ± 0.05
Rn00677549_g1^a^	Exon 10–11	2,427.12 ± 261.53	0.91 ± 0.08	0.97 ± 0.19	0.26 ± 0.07
Rn01447000_m1	Exon 11–12	1.36 ± 0.06	1.47 ± 0.20	1.16 ± 0.22	0.38 ± 0.04

^a^ Cross-reactivity with NMVMs.

### 3.2 Localization of exogenously expressed TRPC1

**FIGURE 3:**
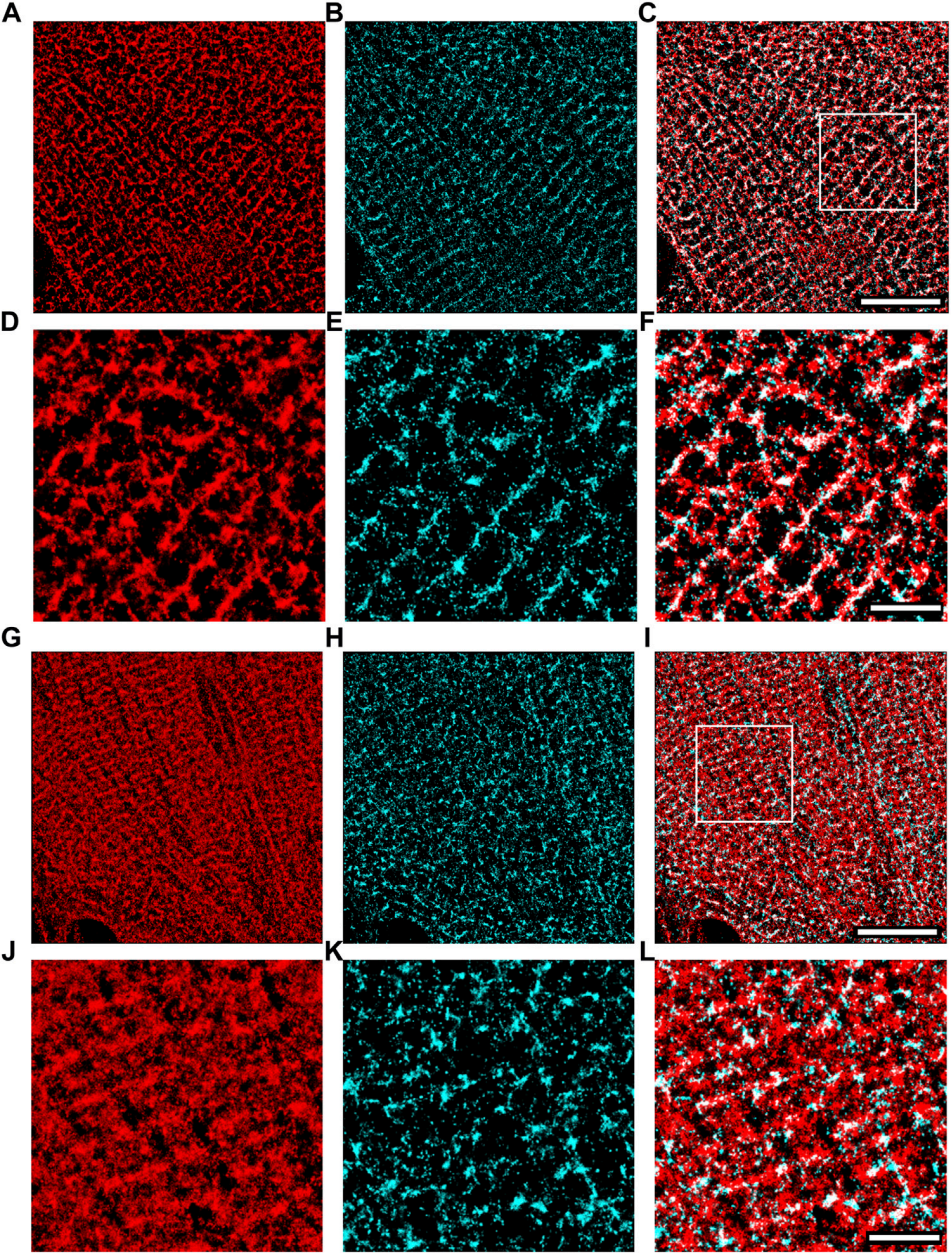
Super-resolution images of NRVMs infected with TRPC1-TagBFP2 **(A–F)** and TagBFP2 **(G**–**L)**. TRPC1-TagBFP2 cells exhibit a striated arrangement of both **(A)** TagBFP2 and **(B)** SERCA2 fluorescence. **(C)** Colocalization of TagBFP2 and SERCA2 in TRPC1-TagBFP2 cells is high, shown in white. **(D–F)** Zoomed in region from **(A–C)** depicted by white box in **(C)**. **(G)** TagBFP2 fluorescence in control cells is distributed throughout the cell, with more dense concentration near sarcomere structures. **(H)** SERCA2 fluorescence in TagBFP2 control cells follows a network, with **(I)** minimal colocalization with TagBFP2. **(J–L)** Zoomed in region from **(G–I)** depicted by white box in **(I)**. Scale bars in **(C,I)** = 10 µm. Scale bars in **(F,L)** = 3 µm.

**FIGURE 4:**
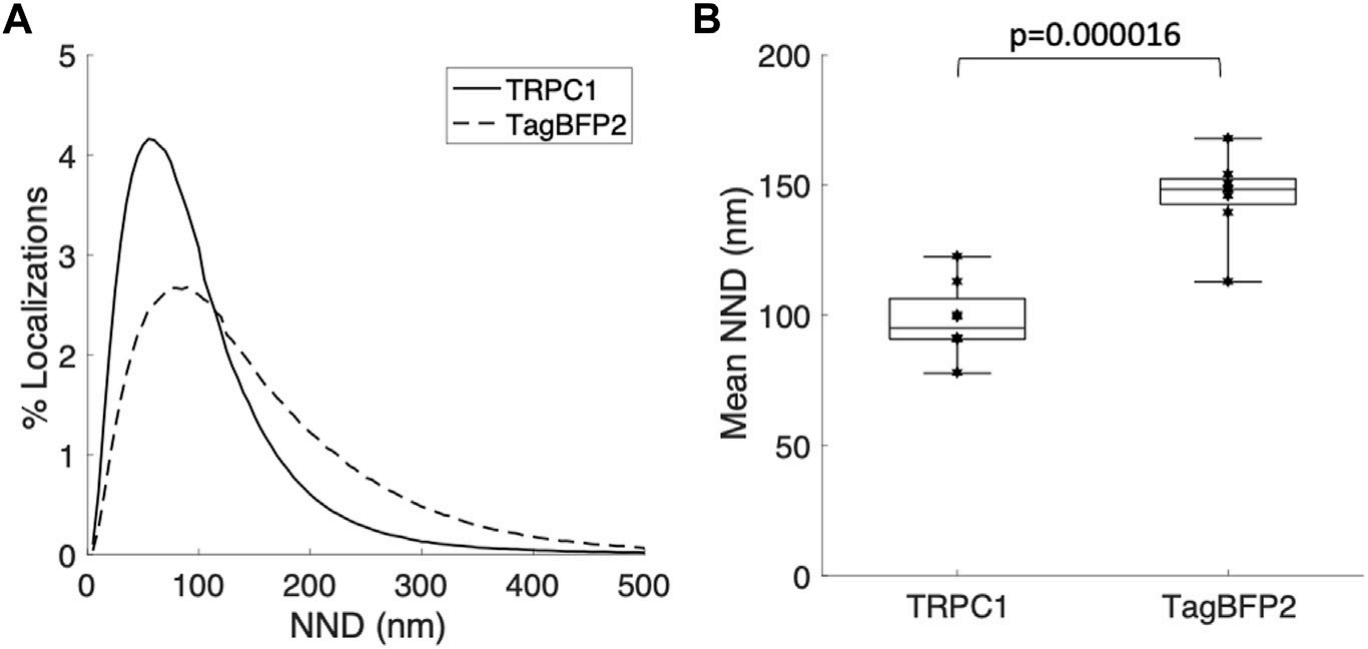
Quantification of colocalization from super-resolution images of NRVMs infected with TRPC1-TagBFP2 or TagBFP2. **(A)** Average distribution of nearest neighbor distances (NND) of SERCA2 from TagBFP2 in TRPC1-TagBFP2 cells and TagBFP2 control cells. **(B)** Mean NND in TRPC1-TagBFP2 cells (98.08 ± 4.98 nm) is significantly lower than in TagBFP2 control cells (145.93 ± 5.55 nm) (*n* = 8 cells). Bracket marks significant difference (*p* < 0.001).

We applied super-resolution imaging to quantify the colocalization of our exogenously expressed TRPC1-TagBFP2 construct with the SR Ca^2+^ pump, SERCA2 (Figure 3). We imaged fixed NRVMs transfected with TRPC1-TagBFP2 and TagBFP2 control cells incubated with antibodies for tRFP, to label the TagBFP2 construct, and for SERCA2, as a marker for SR. TRPC1-TagBFP2 cells exhibit a striated network pattern for both tRFP and SERCA2 antibodies, with abundant overlap in the two signals (Figures 3A–F). In TagBFP2 control cells, tRFP labeling appears throughout the entire cell (Figure 3G). During fixation, the free-floating TagBFP2 tends to get fixed to other structures, and thus it also concentrates at striations of the sarcomeres. However, it appears to be less correlated with the SERCA2 signal (Figure 3H–L). The colocalization of TagBFP2 and SERCA2 was quantified using the nearest neighbor analysis in Vutara SRX software (version 6.05.35). Figure 4A shows the average distribution of nearest neighbor distances from eight images from each cell type. The mean nearest neighbor distance of SERCA2 localization from TagBFP2 localization in TRPC1-TagBFP2 cells is 98.08 ± 4.98 nm, significantly closer than the mean nearest neighbor distance of SERCA2 localization from TagBFP2 localization in TagBFP2 control cells: 145.93 ± 5.55 nm (*p* < 0.001) (Figure 4B).

### 3.3 Expression and localization of GCEPIAer-SNAP adenoviral construct

**FIGURE 5:**
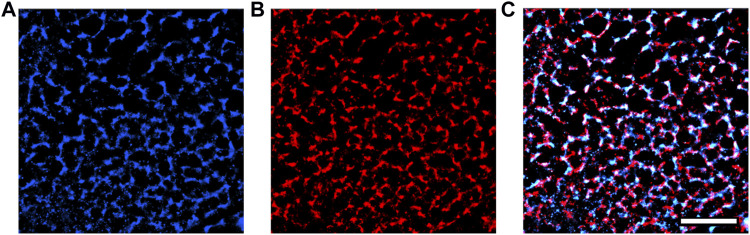
Super-resolution images of GCEPIAer-SNAP construct in TRPC1-TagBFP2 cells. **(A)** SNAP-Cell 647-SiR signal exhibits a network pattern strikingly similar to the **(B)** tRFP signal corresponding to TRPC1-TagBFP2. **(C)** The patterns exhibited from markers for both the GCEPIAer-SNAP construct and TRPC1-TagBFP2 construct reveal abundant overlap. Scale bar = 5 µm.

We also used single-molecule localization microscopy to acquire super-resolution images of our transfected GCEPIAer-SNAP construct (Figure 5). NRVMs infected with both TRPC1-TagBFP2 and GCEPIAer-SNAP were incubated with SNAP-Cell 647-SiR and then fixed and incubated with anti-tRFP to label the TRPC1-TagBFP2 construct. Single-molecule localization microscopy revealed strikingly similar patterns corresponding to the GCEPIAer-SNAP construct and the TRPC1-TagBFP2 construct.

### 3.4 Ca^2+^ concentrations measured *via* fluorescent imaging

We also measured [Ca^2+^]_SR_ in paced NRVMs using fluorescent microscopy. NRVMs cultured on stretchable silicone membranes were infected with GCEPIAer-SNAP and TagBFP2, TRPC1-TagBFP2, or shRNA-TRPC1-TagBFP2. Cells were paced electrically at 0.5 Hz and steady-state pacing fluorescent signals were recorded for cells subject to 0 or 10% sustained stretch for at least 2 min. Fluorescence was calibrated to Ca^2+^ concentration by perforating the membranes with ionomycin and applying solutions with 0 mM Ca^2+^ and 100 mM Ca^2+^ and fitting to the Hill plot curve given by Eq. 1. Scatter plots of measured minimum (*R*_*min*_) and maximum (*R*_*max*_) ratios used for the calibration of each cell are shown in Supplementary Figure S5.

**FIGURE 6:**
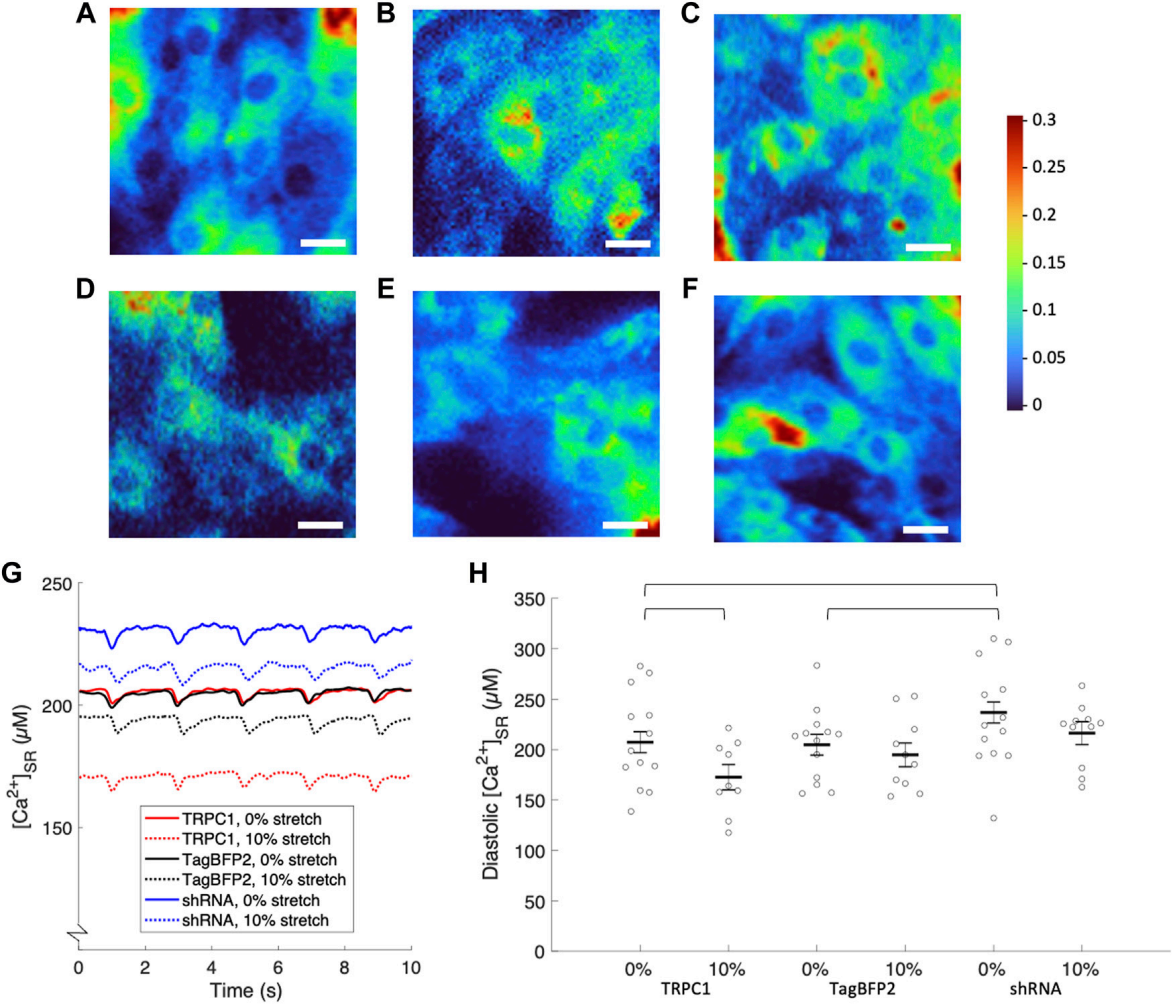
Calibrated [Ca^2+^]_SR_ from fluorescent calcium imaging of NRVMs. Representative ratiometric images of the GCEPIAer signal over the SNAP signal in NRVMs infected with **(A)** TRPC1-TagBFP2 under 0% strain, **(B)** TagBFP2 under 0% strain, **(C)** shRNA-TRPC1-TagBFP2 under 0% strain, **(D)** TRPC1-TagBFP2 under 10% strain, **(E)** TagBFP2 under 10% strain, and **(F)** shRNA-TRPC1-TagBFP2 under 10% strain. Scale bars = 20 µm. **(G)** Example [Ca^2+^]_SR_ transients from TRPC1-TagBFP2, TagBFP2 and shRNA-TRPC1-TagBFP2 infected un stretched and stretched NRVMs. **(H)** Scatter plot of diastolic [Ca^2+^]_SR_ of each infection group with 0 or 10% stretch averaged by membrane; points correspond to the average value of all cells on each membrane and bars represent mean and SEM determined by multilevel mixed-effects model. Diastolic [Ca^2+^]_SR_ in unstretched NRVMs is significantly lower in TRPC1-TagBFP2 and TagBFP2 cells compared to shRNA-TRPC1-TagBFP2 cells. Sustained 10% stretch significantly lowered [Ca^2+^]_SR_ in TRPC1-TagBFP2 infected NRVMs. Brackets mark significant differences (*p* < 0.05).

Figure 6A–F displays representative ratiometric images of GCEPIAer/SNAP in NRVMs infected with TRPC1-TagBFP2, TagBFP2, and shRNA-TRPC1-TagBFP2 under 0 or 10% strain. Example calibrated SR Ca^2+^ transients shown in Figure 6G demonstrate traces representative of the mean from each group for un stretched and stretched NRVMs. Data extracted from the [Ca^2+^]_SR_ traces are displayed in Table 2. Supplementary Figure S6A depicts how data were measured. We measured multiple cells per membrane, so we used a multilevel mixed-effects model to account for clustering and measure the true effect size. Supplementary Figure S7 demonstrates the clustering of cells per membrane. TRPC1-TagBFP2 NRVMs (*p* = 0.045) and TagBFP2 NRVMs (*p* = 0.028) both have significantly lower diastolic [Ca^2+^]_SR_ than shRNA-TRPC1-TagBFP2 (Figure 6H). Likewise, TRPC1-TagBFP2 NRVMs (*p* = 0.045) and TagBFP2 NRVMs (*p* = 0.036) both have significantly lower systolic [Ca^2+^]_SR_ than shRNA-TRPC1-TagBFP2 NRVMs, but no differences were found between the amplitude of the transients of any groups (Supplementary Figures S6B,C).

**TABLE 2:** Data extracted from calibrated [Ca^2+^]SR transients of NRVMs.

Infection	Stretch	# Membranes	# Cells	Diastolic [Ca^2+^]_SR_ (µM)	Systolic [Ca^2+^]_SR_ (µM)	[Ca^2+^]_SR_ release rate (µM/s)	[Ca^2+^]_SR_ reuptake constant s)
TRPC1	0	13	181	207.32 ± 10.41	199.90 ± 10.11	−36.59 ± 3.86	0.21 ± 0.01
TRPC1	10%	9	138	172.64 ± 12.55*	166.43 ± 12.19*	−32.98 ± 4.70	0.24 ± 0.02
TagBFP2	0	13	299	204.78 ± 10.28	198.78 ± 9.98	−35.02 ± 3.78	0.24 ± 0.01
TagBFP2	10%	10	142	194.80 ± 11.84	186.44 ± 11.51	−38.88 ± 4.40	0.24 ± 0.02
shRNA	0	12	237	236.83 ± 10.37	228.53 ± 10.07	−42.97 ± 3.81	0.23 ± 0.01
shRNA	10%	11	134	216.34 ± 11.38	208.43 ± 11.06	−43.89 ± 4.25	0.24 ± 0.02

*Indicates significant difference (*p* < 0.05) from values of the same cell type un stretched.

TRPC1-TagBFP2 NRVMs exhibited significantly reduced diastolic (*p* = 0.033) and systolic [Ca^2+^]_SR_ (*p* = 0.035) when stretched compared to un stretched TRPC1-TagBFP2 NRVMs (Figure 6B, Supplementary Figure S6B). On average, stretch reduced diastolic [Ca^2+^]_SR_ by 16.73% and systolic [Ca^2+^]_SR_ by 16.74% in TRPC1-TagBFP2 infected NRVMs. Diastolic [Ca^2+^]_SR_ in stretched TagBFP2 and stretched shRNA-TRPC1-TagBFP2 NRVMs werenot significantly different from un stretched TagBFP2 (*p* = 0.524) and shRNA-TRPC1-TagBFP2 (*p* = 0.183), respectively. On average, stretch reduced the diastolic [Ca^2+^]_SR_ by 4.87% in TagBFP2 infected NRVMs and 8.65% in shRNA-TRPC1-TagBFP2 infected NRVMs. No significant differences were found for the rate of [Ca^2+^]_SR_ release or the time constant of [Ca^2+^]_SR_ reuptake of any groups (Supplementary Figures S6D,E).

## 4 Discussion

In this study, we provide the first evidence for a role TRPC1 in mechanosensitivity in cardiomyocytes. Using adenoviral transfection, we generated NRVMs with increased and decreased TRPC1 expression. We observed colocalization of TRPC1 constructs with SERCA2, supporting localization of the channel in the SR. Measuring [Ca^2+^]_SR_ directly with a ratiometric genetically encoded SR-targeting Ca^2+^ sensor, we observed a negative correlation between TRPC1 expression and SR Ca^2+^ load. Importantly, stretched TRPC1-overexpressing NRVMs exhibited a decrease in SR Ca^2+^ load vs. control. Together, these results support our hypothesis that TRPC1 forms a mechano-modulated SR Ca^2+^ leak channel. Our findings suggest a new role for TRPC1 in both physiology and pathophysiology in cardiomyocytes.

### 4.1 Confirmation of protein expression of adenoviral constructs in NRVMs

To study the effects of TRPC1 expression on cardiomyocyte Ca^2+^ signaling, we applied adenoviral transfection to generate NRVMs with overexpressed TRPC1 (TRPC1-TagBFP2) and suppressed TRPC1 expression *via* shRNA (shRNA-TRPC1-TagBFP2). NRVMs with TagBFP2 and scrambled shRNA-TagBFP2 expression served as controls. We utilized the TagBFP2 to target our expressed proteins in our analyses. We verified successful expression of our adenoviral constructs using fluorescent microscopy and western blotting (Figure 1, Supplementary Figure S3).

The difference in the adenoviral constructs is apparent in both fluorescent images and western blotting (Figure 1, Supplementary Figure S3). Fluorescence microscopy revealed intracellular, non-nuclear localization of TRPC1-TagBFP2 with less brightness than in TagBFP2, shRNA-TRPC1-TagBFP2, and scrambled shRNA-TagBFP2 NRVMs. The difference in brightness and localization reflects differences in the adenovirus constructs. In the TRPC1-TagBFP2 construct, TagBFP2 is fused to TRPC1, so TagBFP2 fluorescence corresponds to TRPC1 expression and is thus limited by the cell’s limit for protein expression from the construct. The other adenoviral constructs cause TagBFP2 expression under its own promoter and therefore it is expressed at higher concentrations and diffuses within the cell. In western blot, the fused TRPC1-TagBFP2 appears as a band at 96.2 kDa, corresponding to the sum of TRPC1 and TagBFP2. In contrast, western blot from NRVMs infected with control constructs showed bands at 20 and 27 kDa, corresponding to TagBFP2 and its cleaved product. The lack of lower molecular weight bands in TRPC1-TagBFP2 cells further supports that TagBFP2 corresponds exclusively to TRPC1-TagBFP2 expression.

### 4.2 Exogenous TRPC1-TagBFP2 localizes to the SR

Because the fluorescent protein TagBFP2 is fused to TRPC1 in the TRPC1-TagBFP2 construct, the fluorescence corresponding to TagBFP2 reliably localizes the exogenously expressed TRPC1. We implemented single-molecule localization microscopy to measure colocalization of exogenous TRPC1 with the SR Ca^2+^ pump, SERCA2 (Figures 3, 4). Similar to SERCA2, TRPC1-TagBFP2 localizes intracellularly in a regular, striated pattern. It is important to note that NRVMs lack the cell membrane invaginations known as transverse tubules present in adult cardiomyocytes. This reinforces that the patterns in our images must be intracellular. Nearest neighbor analysis revealed high colocalization between TRPC1-TagBFP2 and SERCA2 compared to TagBFP2 and SERCA2 in control cells. This supports our hypothesis that TRPC1 is localized to the SR membrane. Our findings are in agreement with our prior study demonstrating high colocalization of endogenous TRPC1 and SERCA2 (Hu et al., 2020). These findings indicate that both exogenous and endogenous TRPC1 proteins localize similarly in the SR membrane.

Example images in Figure 3 were chosen to best represent the typical patterns seen for TRPC1-TagBFP2 and TagBFP2. SERCA2 appeared with varying degrees of striation throughout our images, likely due to the high variability in NRVMs, varying stages of cell development, and continuous dynamic SR structure remodeling, which is especially prevalent in NRVMs (Vega et al., 2011).

### 4.3 TRPC1 modulates Ca^2+^ leak from SR to cytosol

Single-molecule localization microscopy of the SNAP tag and TagBFP2 fluorescence in TRPC1-TagBFP2 cells revealed matching intracellular network patterns for both GCEPIAer-SNAP and TRPC1-TagBPF2 viral constructs (Figure 5). Proper ER localization of the GCEPIAer protein has been found in studies that first described the protein (Suzuki et al., 2014) and in studies that attached the SNAP tag (Luo et al., 2019). Our images demonstrating the intracellular network pattern also support successful localization of our SR-targeting fluorescent Ca^2+^ sensor, GCEPIAer-SNAP. This sensor was used to evaluate [Ca^2+^]_SR_ transients in NRVMs expressing TRPC1-TagBFP2, TagBFP2, or shRNA-TRPC1-TagBFP2 with either 0 or 10% stretch applied by a uniaxial stretching system. Solutions with 0 Ca^2+^ and 100 mM Ca^2+^ were applied to calibrate Ca^2+^ concentrations from measured fluorescence values.

In the absence of strain, NRVMs infected with shRNA-TRPC1-TagBFP2 exhibited increased diastolic and systolic [Ca^2+^]_SR_ compared to TRPC1-TagBFP2 and TagBFP2 NRVMs (Table 2; Figure 6). This result indicates a decreased Ca^2+^ leak from the SR to the cytosol corresponding to the reduced TRPC1 expression.

We did not detect a significant difference in diastolic and systolic [Ca^2+^]_SR_ measurements between TRPC1-TagBFP2 and TagBFP2 cells in the absence of strain. We suggest that this is at least in part explained by large variability of our measurements on NRVMs. However, in contrast to these results, our prior study on NRVMs demonstrated a significant difference in [Ca^2+^]_SR_ load in TRPC1 vs. control unstrained myocytes (Hu et al., 2020). A major difference in the experimental design of the prior study and this one may help explain the difference in results. In the prior study, we evaluated [Ca^2+^]_SR_ load in an instant after a 2 min period of rest, while in the current study we measured [Ca^2+^]_SR_ in real time in actively pacing myocytes. It is possible that in pacing myocytes, effects of increased SR leak by TRPC1 overexpression are to some degree attenuated by increased SERCA uptake and NCX extrusion. We hypothesize that the small difference in leak due to TRPC1 overexpression can only be observed in quiescent cells, when larger sarcolemmal currents are negligible, as in our prior experiments (Hu et al., 2020).

In NRVMs under sustained 10% stretch, TRPC1-TagBFP2 cells exhibited decreased diastolic and systolic [Ca^2+^]_SR_ compared to unstretched TRPC1-TagBFP2 cells by almost 17%. These findings suggest a positive correlation between TRPC1 channel activation by stretch and SR to cytosol Ca^2+^ leak, which we propose implicates TRPC1 channels as mechano-modulated SR Ca^2+^ leak channels. Our findings fill a gap in understanding the components of SR Ca^2+^ leak. Most studies on SR leak explain leak only as a function of ryanodine receptors (RyR) in the form of localized release events known as sparks. However, it was demonstrated over a decade ago that total leak also includes a non-spark and a non-RyR component (Santiago et al., 2010; Zima et al., 2010). While over 20 different proteins have been proposed to underlie endoplasmic reticulum Ca^2+^ leak, the major contributors are still not well understood (Lemos et al., 2021).

We did not detect any statistical differences between experimental groups for the [Ca^2+^]_SR_ transient amplitude [Ca^2+^]_SR_ release rate, nor [Ca^2+^]_SR_ reuptake constant (Supplementary Figure S6). While we expected to measure a difference in the release rate and reuptake constant due to changes in SR leak, it is possible other mechanisms underlying Ca^2+^ handling attenuate effects on overall calcium flux through the SR membrane. It is also possible that the differences are much smaller vs. the variation in our experiments and thus undetectable. Also, Ca^2+^ buffering and saturation of GCEPIAer-SNAP could affect our results. Binding kinetics were characterized by (Luo et al., 2019). Our measurements are below the dissociation constant of 514 µM so issues with saturation are unlikely. However, we cannot exclude a minor effect of buffering on calcium dynamics. Furthermore, we lack quantitative information on the dynamics of Ca^2+^ binding and fluorescence responses to changes of SR Ca^2+^ concentrations, thus our assessment of fast processes such a Ca^2+^ release could be limited.

### 4.4 The debate of TRPC1 mechanosensitivity

While our results imply a role in mechanotransduction, we did not decipher whether TRPC1 is directly activated or indirectly modulated downstream of other mechanosensors. In this study, we applied a 10% uniaxial stretch to the NRVMs. We confirmed consistent membrane strain along the applied axis (data not shown). Neonatal myocytes, unlike adult myocytes, do not yet have an established longitudinal axis. Instead, the sarcomeres are oriented in various directions. Thus, the uniaxial stretch applied by our stretching system results in heterogeneous strain orientations and magnitudes throughout the cells and their organelles. This is important to consider when discussing the mechanism for TRPC1 activation and quantifying strain effects.

When TRPC1 was first suggested to form mechanosensitive channels, it was thought to be gated by tension in the lipid bilayer of frog oocytes (Maroto et al., 2005). Contrastingly, direct activation of mammalian TRP channels was recently refuted (Nikolaev et al., 2019). Nikolaev et al. reconstituted 11 mammalian TRP channels into HEK293T cells and found that none of them exhibited increased channel activity when exposed to tension applied *via* negative pipette pressure through a patch-clamp. While TRPC1 was not studied, the TRPC family members TRPC3, TRPC5, and TRPC6 were (Nikolaev et al., 2019). It is possible that TRPC1 functions as its family members and activity isn’t directly related to membrane tension. However, we cannot rule out that TRPC1 has different mechanosensitive properties.

Although controversy surrounds direct TRPC gating by membrane tension, another possibility for direct mechanical gating is through tethering to the cytoskeleton. A TRP family member, No mechanoreceptor potential C (NOMPC), was found to be mechanically gated by a mechanism dependent on tethering of 29 intracellular ankyrin repeats to microtubules (Zhang et al., 2015). It was suggested that the mechanism for this activation is through compression of the ankyrin repeats, which act as a spring to “push” open the channel pore, rather than pulling (Wang et al., 2021). Wang et al. (2021) demonstrate that the channel’s open probability increases with positive pipette pressure, rather than negative pipette pressure in patch-clamp. If TRPC channels use a similar mechanism for mechanogating, this could explain the contrasting reports that primarily study the channels using membrane tension or negative pipette pressure, as these were shown to reduce the pore size of NOMPC. TRPC1, however, only has four ankyrin repeats as compared to the 29 of NOMPC. Based on this hypothesis, a more appropriate model of mechanical intervention that focuses on compression should be considered than uniaxial stretch as we performed here. However, in consideration of the uniaxial stretch we applied, this would also result in compression of the cell along other axes, which could explain the changes we measured. The question of direct TRPC1 mechanosensitivity brings up limitations in our experimental design. If the TRPC1 were directly mechanosensitive, the activated current should correlate to the intensity of the mechanical stimulus. Unfortunately, due to the highly variable nature of NRVMs and their viability, our study was limited to a single applied strain. We chose to apply 10% strain because it is close to physiological strain in the *in-situ* heart (Caporizzo and Prosser, 2021). Initial work showed that this amount of strain can be reliably applied with the Strex stretching system. Higher magnitudes of strain corresponded to shifting of the membrane on the device and cells drifting out of focus (experiments not shown).

Alternatively, it is possible that TRPC1 isn’t a primary mechanosensor, but follows downstream mechanosensory signaling cascades, as suggested for TRPC6 (Mederos y Schnitzler et al., 2008; Nikolaev et al., 2019). A number of G protein-coupled receptors (GPCRs) were found to be mechanosensitive (Storch et al., 2012). For example, several studies demonstrate that the G_q,11_ protein-coupled angiotensin II type-1 receptor (AT_1_R) is a mechanosensitive receptor, capable of being activated by stretch without ligand binding, and has a role in inducing cardiac hypertrophy (Zou et al., 2004; Rakesh et al., 2010). TRPC channels were shown to be activated by second messengers generated by AT_1_R activation (Mederos y Schnitzler et al., 2008; Seth et al., 2009). Therefore, TRPC1 channel activation may occur downstream of this mechanically induced GPCR activation. This would indicate that TRPC1 channels are only indirectly mechanosensitive. However, TRPC1 and TRPC6 are thought to be activated *via* different second messengers along downstream pathways of AT_1_R activation (Saleh et al., 2006), which indicates a need to decipher the roles of TRPC1 and AT_1_R mechanosensation.

### 4.5 Implications of mechano-modulated TRPC1 SR Ca^2+^ leak in cardiac physiology and pathophysiology

Regardless if TRPC1 Ca^2+^ leak is directly or indirectly modulated by stretch, our finding has both physiological and pathophysiological implications for cardiomyocytes. Our results suggest that TRPC1 contributes to a Ca^2+^ leak from the SR that is augmented by mechanical deformations. Given these results, we postulate that the physiological roles of TRPC1 channels include preventing SR Ca^2+^ overload, similar to that described for RyR-mediated leak (Shannon et al., 2002). This is critical since mechanical strains are also associated with a Ca^2+^ influx through the sarcolemma. In the absence of an augmented leak as a balancing mechanism, increased SERCA activity in response to the influx of extracellular Ca^2+^ would result in an overloaded SR. A role in regulating excess Ca^2+^ in the SR complements its well-established role in store-operated calcium entry, as TRPC1 could contribute to the entire loop of regulation of Ca^2+^ depleted and overloaded SR.

Another physiological role of mechano-modulated TRPC1 channels could be in maintaining ventricular wall tension in response to increased pressure in the ventricles. An increase in pressure causes a strain on cardiomyocytes, which warrants the cells to respond with an equal increase in contractile tension to maintain structural integrity. If TRPC1 is mechano-modulated, directly or indirectly, it could have a role in supplying the cell with additional Ca^2+^ necessary to maintain the muscular tone. When the heart experiences an increased pressure and cardiomyocytes stretch, increased TRPC1 leak would shift Ca^2+^ from the SR to the cytosol. Depending on the temporal dynamics of this shift, this could support excitation-contraction coupling and help to maintain ventricular wall tension in response to the increased load. Under chronically sustained strain conditions such as expected for hypertension, this same mechanism has the potential to contribute to pathological development. If expression and leak through TRPC1 is increased and not compensated by SERCA activity, the SR Ca^2+^ load could become diminished leading to reduced Ca^2+^ release, force development and contraction.

In our prior work, we explored the impact of TRPC1-mediated SR Ca^2+^ leak on myocyte electrophysiology (Hu et al., 2020; Streiff and Sachse, 2022). In particular, we investigated the relationship between SR leak and the action potential using a computational model of rabbit ventricular myocytes (Hu et al., 2020). SR Ca^2+^ leak did not significantly affect resting membrane potential and exhibited only a marginal positive correlation with action potential duration. In another *in situ* study of a human ventricular myocyte, modulating SR Ca^2+^ leak had negligible effects on both resting membrane potential and action potential duration at any pacing frequency (Streiff and Sachse, 2022). It seems unlikely that TRPC1 leak has a role in modulating myocyte electrophysiology in physiological conditions. However, we cannot exclude such a role in disease, e.g., after upregulation of the TRPC1 expression in hypertrophy.

As a body of literature covers its involvement in cardiac hypertrophy, the role of TRPC1 in maladaptive cardiac remodeling can be reviewed in the context of our results here. Under chronic pressure overload conditions, TRPC1 leak increases. Many studies show that TRPC1 expression is elevated in pressure overload models (Ohba et al., 2007; Seth et al., 2009; Morine et al., 2016). The fact that suppression or deletion of the TRPC1 gene is significant to lessen the degree of maladaptive remodeling suggests that increased TRPC1 leak in disease is not just the result of the change in conditions, but that it plays a role contributing to remodeling (Ohba et al., 2007; Seth et al., 2009; Tang et al., 2019). This conclusion is opposite to what has been suggested about RyR leak (Mohamed et al., 2018). The Ca^2+^/calcineurin/nuclear factor of activated T cells (NFAT) pathway is well-established as a signaling cascade responsible for hypertrophic remodeling. NFAT operates as an integrator of cumulative Ca^2+^ (Hannanta-Anan and Chow, 2016). Indeed, chronically increased leak through TRPC1 channels in disease could contribute to the elevated cumulative Ca^2+^ necessary to trigger NFAT translocation to the nucleus and trigger hypertrophic remodeling.

Another potential pathologic consequence of chronically increased SR leak through TRPC1 channels is increased susceptibility to arrhythmia. As has been discussed, primarily in the context of RyR leak, chronically increased SR leak makes cardiomyocytes increasingly susceptible to arrhythmias (Hannanta-Anan and Chow, 2016; Siri-Angkul et al., 2021; Streiff and Sachse, 2022). Indeed, recent studies suggested a link between TRPC-mediated currents and arrhythmogenesis (Wen et al., 2018; Siri-Angkul et al., 2021). Future studies could investigate arrhythmogenic potential in TRPC1-overexpressing cells including measurements of other Ca^2+^ currents known to contribute to arrhythmogenesis, such as NCX and SERCA.

We acknowledge important limitations of translation of our findings in rodent cultured neonatal to adult cardiomyocytes in larger mammals including human. We used NRVMs because reliable protocols for adenoviral infection and expression of construction have been established. Beyond differences due to culturing, e.g., on gene expression and myocyte shape, we note age-specific difference of the contribution of sarcolemmal and SR fluxes to the Ca^2+^ transients. Prior work on rat revealed that freshly isolated NRVMs exhibit a much larger contribution of sarcolemmal vs. SR fluxes to the Ca^2+^ transient that adult cardiomyocytes (Chin et al., 1990). We suggest that these differences are reflected in the low SR Ca^2+^ content and small degree of Ca^2+^ release in our studies vs. prior studies on adult cardiomyocytes. We also note differences in calcium signaling in rodents and larger mammals, which limit our conclusions. It will be critical to confirm a role of TRPC1 channels in mechano-modulated SR Ca^2+^ leak in adult cardiomyocytes, both in isolation and in tissues, as well as in species beyond rodent.

### 4.6 RT-qPCR reveals endogenous and exogenous TRPC1 expression changes

We used RT-qPCR to measure TRPC1 expression changes in our transfected cells. We tested five rat TRPC1 primers, one spanning each of exon 3-4 (Rn00677552_m1), 6-7 (Rn00677554_m1), 8-9 (Rn00585625_m1), 10-11 (Rn00677549_g1), and 11-12 (Rn01447000_m1). There was a reasonably consistent reduction in expression in the shRNA-TagBFP2 group in each tested primer (Table 1; Figure 2). None of the tested primers show significant differences between the TagBFP2 and scrambled shRNA-TagBFP2 controls. These results confirm that transfection of our shRNA-TRPC1-TagBFP2 construct does decrease expression of TRPC1.

We found varying degrees of cross-reactivity with mouse TRPC1 in the tested primers (Supplementary Table S2). Both exon 3-4 and exon 10-11 appeared to cross-react with endogenous mouse TRPC1. In TRPC1-TagBFP2 NRVMs, RT-qPCR for these two primers showed a 178-fold increase in expression for exon 3-4 and a 2427-fold increase for exon 10-11. Since these primers react with both mouse and rat TRPC1, these values are a sum of exogenously expressed mouse TRPC1 and endogenously expressed rat TRPC1. These results demonstrate high expression of exogenous mouse TRPC1 from our transfection, supporting imaging and western blot results. The tested primers for exon 6-7 and exon 8-9 did not exhibit cross-reactivity with WT NMVMs (Supplementary Table S2), and the associated TRPC1-TagBFP2 NRVMs exhibit similar fold-change expression from TagBFP2 NRVMs of 8.8 and 7.7-fold change, respectively (Table 1). This increase in expression likely indicates an increase of endogenous rat TRPC1 separate from our exogenously infected mouse TRPC1, supporting the existence of a positive feedback loop for TRPC1 gene expression.

In summary, several primers show cross-reactivity with mouse, demonstrating increased exogenous mouse TRPC1, and several do not exhibit cross-reactivity, demonstrating increased endogenous rat TRPC1. However, the difference in expression changes within each category varies (Table 1; Figure 2). Interestingly, expression changes were not detected in TRPC1-TagBFP2 cells for exon 11–12. The difference in mRNA expression levels across primers hints at the differential expression of splice variants. Splice variants in TRPC1 gene expression have been preliminarily characterized in vascular smooth muscle cells and human aorta mRNA, with known cassette exons 2, 3, 5, 6, 7, 8, and 9, each of which can be alternatively spliced, and exon 4, which can be constitutively spliced (Dedman et al., 2011; Dietrich et al., 2014). While not a focus of our study, these findings suggest further exploration with more specific primers will improve understanding of the variants of TRPC1 expressed in cardiomyocytes.

## Data Availability

The raw data supporting the conclusion of this article will be made available by the authors, without undue reservation.
