# Supplementary material for: TRPC1 channels underlie stretch-modulated sarcoplasmic reticulum calcium leak in cardiomyocytes
Source: Front Physiol. 2022 Dec 23;13:1056657. doi: 10.3389/fphys.2022.1056657 (PMC9817106; doi:10.3389/fphys.2022.1056657)
Supplement: Supplementary file 1 [file DataSheet1.PDF]

Supplementary Material

Supplementary Tables

Supplemental Table S1. Quantity, purity, and integrity of RNA and cDNA stocks for RT-qPCR determined by spectrophotometry

Sample	RNA ng/µl	RNA purity A260/A280	cDNA ng/µl	cDNA purity A260/A280
WT NMVM litter 1.1	15.6	2.02	1970.8	1.91
WT NMVM litter 1.2	13.8	2.03	2121.5	1.91
WT NRVM litter 1	64.0	2.07	2343.8	1.92
WT NRVM litter 2	55.6	2.02	2284.7	1.89
TRPC1-TagBFP2 litter 1	40.3	2.02	2566.7	1.86
TagBFP2 litter 1	16.8	1.99	2172.0	1.92
Scrambled shRNA-TagBFP2 litter 1	51.3	2.00	2054.1	1.92
shRNA-TRPC1-TagBFP2 litter 1	47.8	2.03	2247.1	1.91
TRPC1-TagBFP2 litter 2	44.1	2.05	2214.8	1.91
TagBFP2 litter 2	45.6	2.03	2281.1	1.91
Scrambled shRNA-TagBFP2 litter 2	42.3	2.05	2336.0	1.90
shRNA-TRPC1-TagBFP2 litter 2	32.2	2.05	2225.9	1.92
TRPC1-TagBFP2 litter 3	63.8	2.05	2128.8	1.92
TagBFP2 litter 3	60.7	2.05	2372.2	1.90
Scrambled shRNA-TagBFP2 litter 3	79.7	2.06	2352.0	1.89
shRNA-TRPC1-TagBFP2 litter 3	76.0	2.02	2138.5	1.92

Supplemental Table S2. RT-qPCR ΔCt from reference gene 18s of WT NRVM and NMVM

Primer	WT NRVM litter 1	WT NRVM litter 2	WT NMVM litter 1.1	WT NMVM litter 1.2
Rn00677552_m1	19.00	18.03	24.16	24.52
Rn00677554_m1	19.31	18.32	-	-
Rn00585625_m1	19.17	17.70	-	-
Rn00677549_g1	19.04	17.79	19.19	19.48
Rn01447000_m1	18.06	17.51	-	-

Supplementary Figures

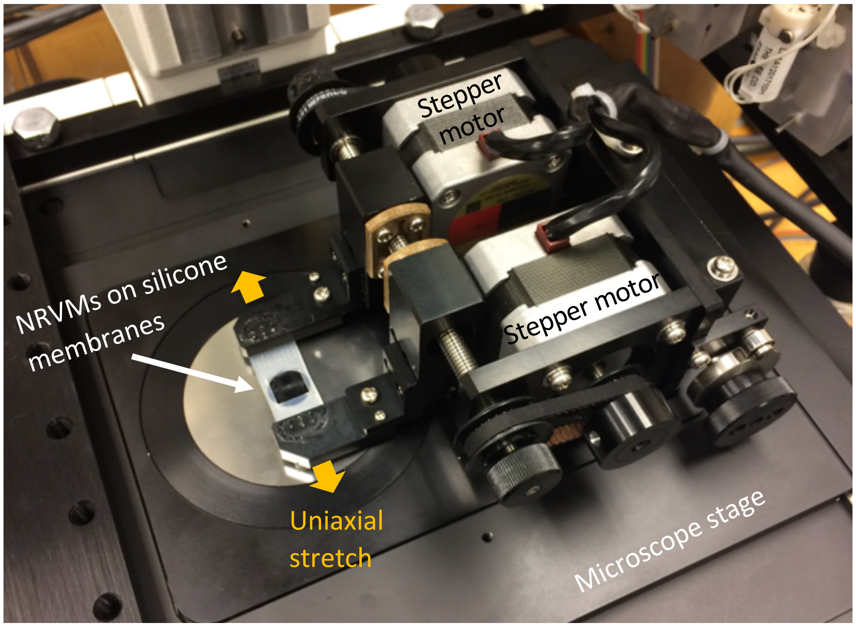

Supplemental Figure S1. Uniaxial stretching system (STREX STB-150W) mounted on our microscope stage. NRVMs are cultured on silicone membranes and held in place with small pins on 2 arms. The system applies stepper motors to uniaxially stretch the silicone chambers according to the programmed distance and frequency. We programmed the system to stretch the silicone membranes 10% in 0.5 s and then hold the membrane in sustained stretch until the experiment was completed.

```
 * ----- begin analysis of calibratedcasrs -----
cd "/net/woody.cvrti.utah.edu/Volumes/confocaldata/TRPC_GCEPIA/Stata/"

use final,clear

graph box calibratedcasrs, over(stretch) over(infection2)

set showbaselevels on

* use cooksd to find influential observations
regress calibratedcasrs ib0.stretch##ib2.infection2
capture drop d
predict d, cooksd
tab d if d>4/_N

capture drop outlier
gen outlier=0
replace outlier=1 if d>4/_N
tab outlier
graph box calibratedcasrs if outlier==0, over(stretch) over(infection2)

cls
mixed calibratedcasrs ib0.stretch##ib2.infection2 if outlier==0|| membraneid:
margins stretch infection2 stretch#infection2
margins stretch infection2 stretch#infection2, post coeflegend

lincom _b[1.stretch#1bn.infection2]-_b[0bn.stretch#1bn.infection2]
  // TRPC1stretched - TRPC1
lincom _b[1.stretch#2.infection2]-_b[0bn.stretch#2.infection2]
  // BFPstretched - BFP
lincom _b[1.stretch#3.infection2]-_b[0bn.stretch#3.infection2]
  // shRNAstretched - shRNA

lincom _b[0bn.stretch#1bn.infection2]-_b[0bn.stretch#3.infection2]
  // TRPC1 - shRNA
lincom _b[0bn.stretch#3.infection2]-_b[0bn.stretch#2.infection2]
  // shRNA - BFP
lincom _b[0bn.stretch#1bn.infection2]-_b[0bn.stretch#2.infection2]
  // TRPC1 - BFP

  drop if outlier==1

    collapse (mean) calibratedcasrs,by(membraneid)

 * ----- end analysis of calibratedcasrs -----
```

Supplemental Figure S2. Example of Stata code used for statistical analysis of $[Ca^{2+}]_{SR}$ transient measurements. The above code tests the calibrated values of systolic $[Ca^{2+}]_{SR}$ but the same code was used for all other measurements. First, influential observations were filtered out using cooksd and then a multilevel mixed-effects linear regression was used to account for the true effect size based on cells measured on the same membrane; linear combinations were used to analyze statistical differences between groups.

A

B

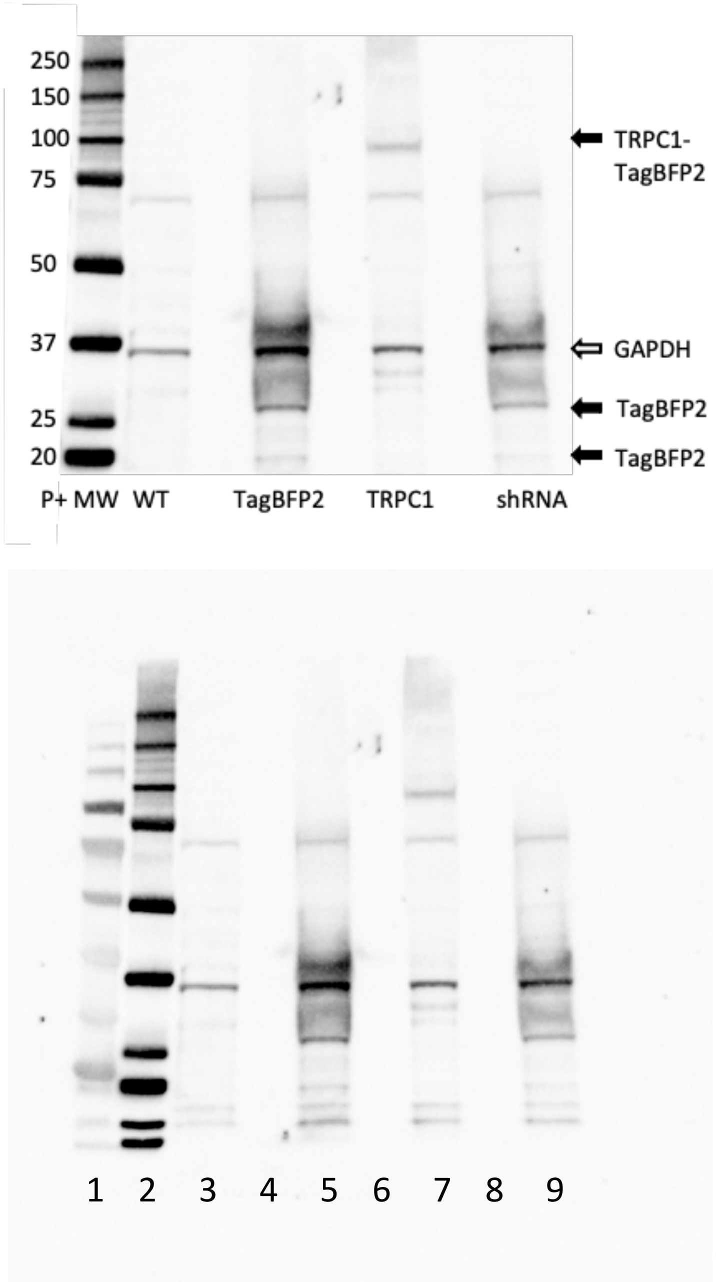

Supplemental Figure S3. Western blot of infected NRVMs with antibodies for tRFP (AB233) and GAPDH (AB8245). (**A**) Annotated western blot targeting tRFP (AB233) reveals bands corresponding to TagBFP2 at 20 and 27 kDa in TagBFP2 and shRNA-TRPC1-TagBFP2 NRVMs. The band at 96.2 kDa corresponding to the weight of TRPC1 and TagBFP2 together is present only in the TRPC1-TagBFP2 sample. Housekeeping gene GAPDH (AB8245, white arrow) appears at 36 kDa in all samples. (**B**) The uncropped and unannotated western blot corresponding to (**A**). Lane 1: Blue MW, Lane 2: P+ MW, Lane 3: WT NRVMs, Lane 4: blank, Lane 5: TagBFP2 infected NRVMs, Lane 6: blank, Lane 7: TRPC1-TagBFP2 infected NRVMs, Lane8: blank, Lane 9: shRNA-TRPC1-TagBFP2 infected NRVMs.

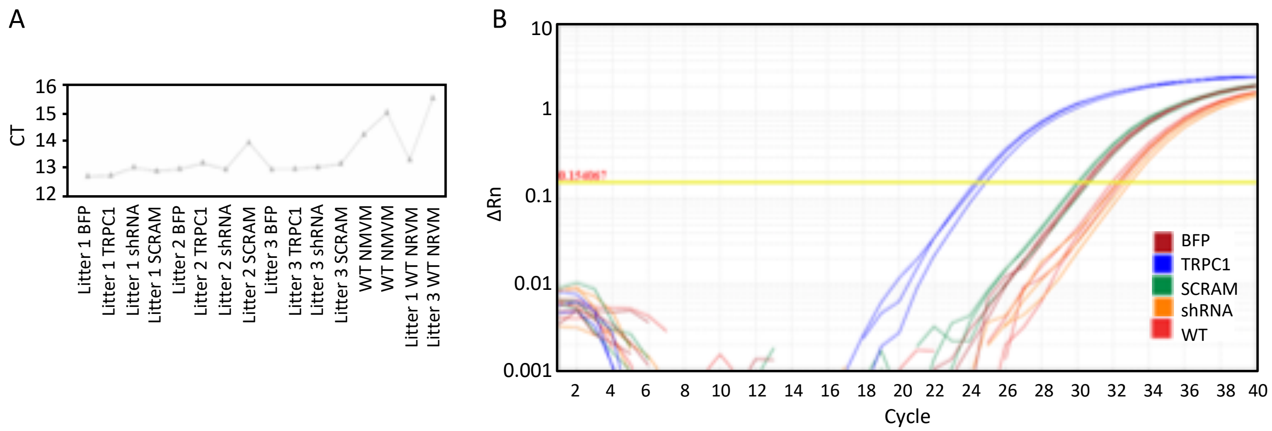

Supplemental Figure S4. (**A**) Profile of endogenous control used for RT-qPCR, 18S, across all samples. (**B**) Example amplification plot of litter 1 for RT-qPCR primer targeting exon 3-4 (Rn00677552_m1).

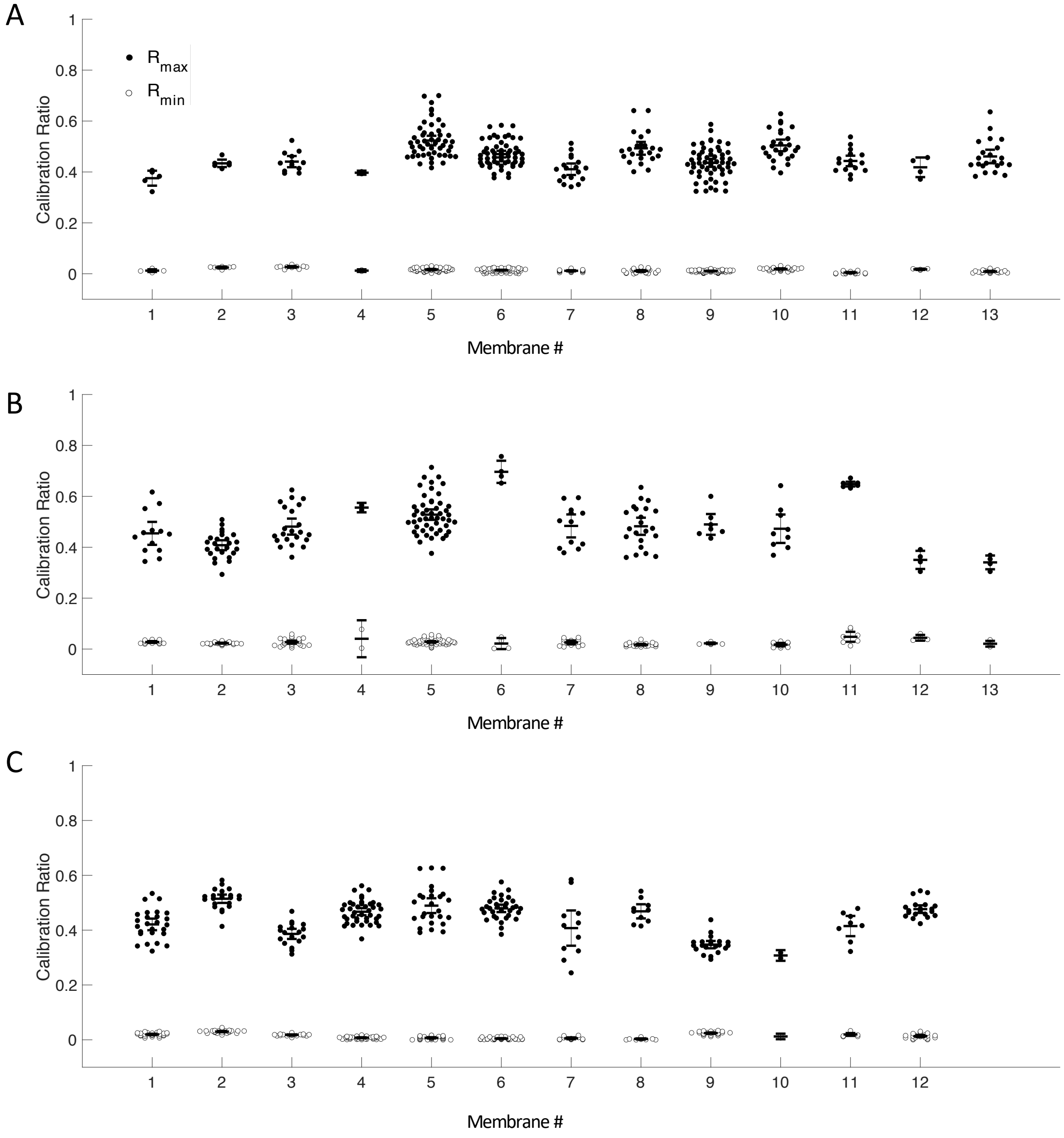

Supplemental Figure S5. Scatter plots of the minimum (R_{min}) and maximum (R_{max}) ratios (GCEPIAer/SNAP) measured in 0 mM Ca^{2+} and 100 mM Ca^{2+} solutions, respectively. Every point represents measurements from NRVMs infected with (**A**) TRPC1-TagBFP2, (**B**) TagBFP2, and (**C**) shRNA-TRPC1-TagBFPs.

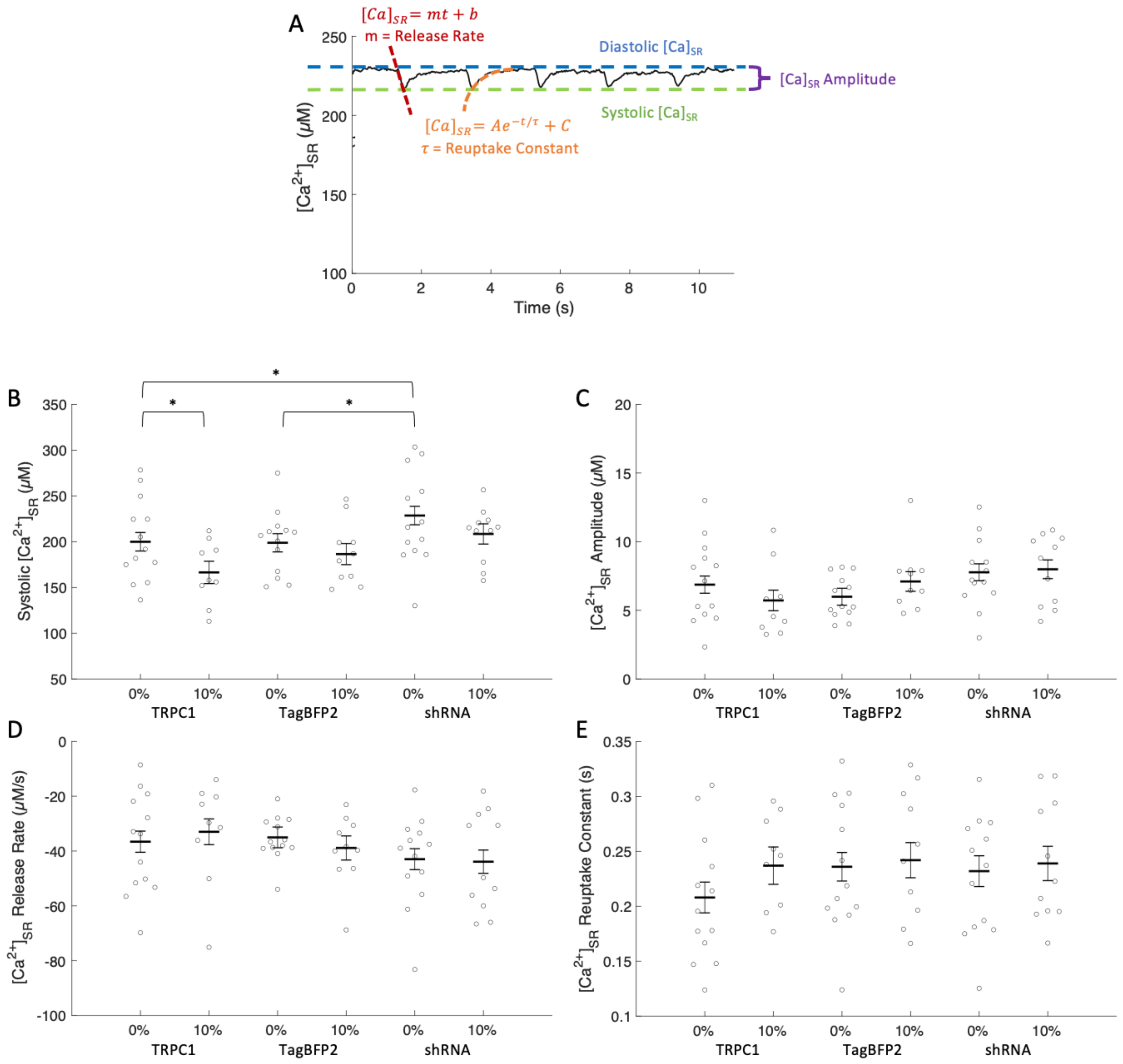

Supplemental Figure S6. Additional measurements from calibrated $[Ca^{2+}]_{SR}$ from fluorescent Ca^{2+} imaging of TRPC1-TagBFP2, TagBFP2 and shRNA-TRPC1-TagBFP2 infected NRVMs. (**A**) Depiction of measurements obtained from each transient on a representative $[Ca^{2+}]_{SR}$ trace. (**B**) Systolic $[Ca^{2+}]_{SR}$ in unstretched NRVMs is significantly lower in TRPC1-TagBFP2 and TagBFP2 cells compared to shRNA-TRPC1-TagBFP2 cells. Sustained 10% stretch significantly lowered $[Ca^{2+}]_{SR}$ in TRPC1-TagBFP2 infected NRVMs. Brackets mark significant differences ($p<0.05$). (**C**) $[Ca^{2+}]_{SR}$ amplitude, (**D**) release rate, and (**E**) reuptake time constant show no significant differences across groups. Scatter plots display measurements of each infection group with 0 or 10% stretch averaged by membrane; bars represent mean and SEM determined by multilevel mixed-effects model.

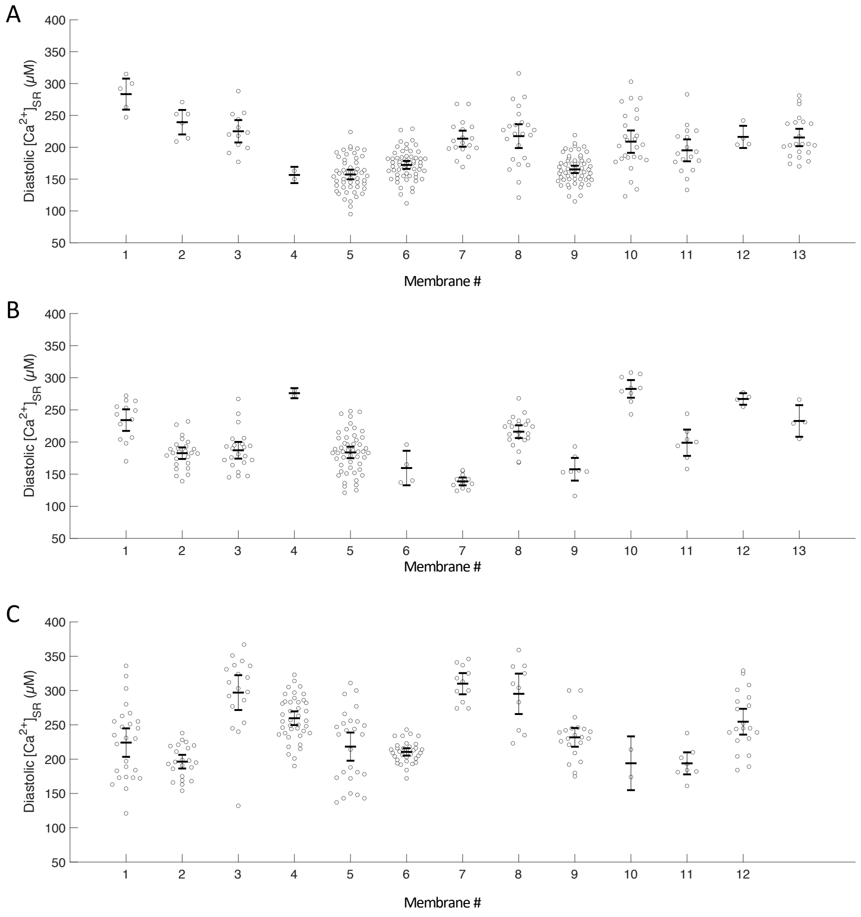

Supplemental Figure S7. Clustering variability across membranes. Scatter plots of diastolic $[Ca^{2+}]_{SR}$ measured for each cell per membrane of NRVMs infected with (A) TRPC1-TagBFP2, (B) TagBFP2, and (C) shRNA-TRPC1-TagBFP2. Bars represent the mean and SEM of measurements on each membrane.
